# The plasma glutamate concentration as a complementary tool to differentiate benign PET-positive lung lesions from lung cancer

**DOI:** 10.1186/s12885-018-4755-1

**Published:** 2018-09-03

**Authors:** K. Vanhove, P. Giesen, O. E. Owokotomo, L. Mesotten, E. Louis, Z. Shkedy, M. Thomeer, P. Adriaensens

**Affiliations:** 1 Faculty of Medicine and Life Sciences, Hasselt University, Martelarenlaan 42, B-3500 Hasselt, Belgium; 2 Department of Respiratory Medicine, Algemeen Ziekenhuis Vesalius, Hazelereik 51, B-3700 Tongeren, Belgium; 3 Institute for Biostatistics and Statistical Bioinformatics, Hasselt University, Agoralaan Building D, B-3590 Diepenbeek, Belgium; 4 Department of Nuclear Medicine, Ziekenhuis Oost-Limburg, Schiepse Bos 6, B-3600 Genk, Belgium; 5 Department of Respiratory Medicine, University Hospital Leuven, Herestraat 49, B-3000 Leuven, Belgium; 6 Department of Respiratory Medicine, Ziekenhuis Oost-Limburg, Schiepse Bos 6, B-3600 Genk, Belgium; 7 Applied and Analytical Chemistry, Institute for Materials Research, Hasselt University, Agoralaan Building D, B-3590 Diepenbeek, Belgium

**Keywords:** Lung cancer, Lung inflammation, ^1^H-NMR, Metabolic phenotype, Glutamate, ROC

## Abstract

**Background:**

Pulmonary imaging often identifies suspicious abnormalities resulting in supplementary diagnostic procedures. This study aims to investigate whether the metabolic fingerprint of plasma allows to discriminate between patients with lung inflammation and patients with lung cancer.

**Methods:**

Metabolic profiles of plasma from 347 controls, 269 cancer patients and 108 patients with inflammation were obtained by ^1^H-NMR spectroscopy. Models to discriminate between groups were trained by PLS-LDA. A test set was used for independent validation. A ROC curve was built to evaluate the diagnostic performance of potential biomarkers.

**Results:**

Sensitivity, specificity, PPV and NPV of PET-CT to diagnose cancer are 96, 23, 76 and 71%. Metabolic profiles differentiate between cancer and inflammation with a sensitivity of 89%, a specificity of 87% and a MCE of 12%. Removal of the glutamate metabolite results in an increase of MCE (38%) and a decrease of both sensitivity and specificity (62%), demonstrating the importance of glutamate for discrimination. At the cut-off point 0.31 on the ROC curve, the relative glutamate concentration discriminates between cancer and inflammation with a sensitivity of 85%, a specificity of 81%, and an AUC of 0.88. PPV and NPV are 92 and 69%. In PET-positive patients with a relative glutamate level ≤ 0.31 the sensitivity to diagnose cancer reaches 100% with a PPV of 94%. In PET-negative patients, a relative glutamate level > 0.31 increases the specificity of PET from 23% to 58% and results in a high NPV of 100%. In case of discrepancy between SUV_max_ and the glutamate concentration, lung cancer is missed in 19% of the cases.

**Conclusion:**

This study indicates that the ^1^H-NMR-derived relative plasma concentration of glutamate allows discrimination between lung cancer and lung inflammation. A glutamate level ≤ 0.31 in PET-positive patients corresponds to the diagnosis of lung cancer with a higher specificity and PPV than PET-CT. Glutamate levels > 0.31 in patients with PET negative lung lesions is likely to correspond with inflammation. Caution is needed for patients with conflicting SUV_max_ values and glutamate concentrations. Confirmation is needed in a prospective study with external validation and by another analytical technique such as HPLC-MS.

**Electronic supplementary material:**

The online version of this article (10.1186/s12885-018-4755-1) contains supplementary material, which is available to authorized users.

## Background

Lung cancer is the leading cause of cancer death in men and the second leading cause of cancer death in women worldwide [1]. It was estimated that 1.8 million new lung cancer cases and 1.6 million lung cancer death occurred in 2012 worldwide, accounting for almost 19% of all cancer deaths [2].

Most patients with lung cancer are diagnosed with advanced disease, resulting in a very low global 5-year survival of only 18% [3]. Screening aims to detect lung cancer in an early stage, before patients experience clinical symptoms, and when treatment is the most effective. The principal aim of screening for lung cancer by low-dose computed tomography (CT) is to reduce lung cancer-specific death [4, 5]. CT-imaging often identifies suspicious pulmonary nodules or focal lung lesions, but cannot verify whether these are the results of benign disease or a truly aggressive malignancy, leading to supplementary imaging techniques or additional CT scans with cumulative radiation levels or invasive procedures, such as tissue biopsies [4, 6].

Due to limitations of radiological imaging techniques in the differentiation between benign and malignant tissue, positron emission tomography (PET) has become an additional option for the evaluation of suspicious pulmonary nodules and other focal lung lesions [7].

Unlike normal tissue, malignant tumors are characterized by an increased glycolysis, which leads to an elevated glucose uptake. ^18^F-fluorodeoxyglucose (^18^F-FDG) PET-CT makes use of this characteristic in order to diagnose and stage various human malignancies [8–10]. The standardized uptake value (SUV) is a semi-quantitative measurement of the tissue ^18^F-FDG accumulation rate [10]. The maximal standardized uptake value (SUV_max_) is the voxel with the highest ^18^F-FDG uptake value in the region of interest.

However, regardless of its high accuracy and sensitivity, high ^18^F-FDG uptake is not cancer-specific. High levels of ^18^F-FDG uptake can also be detected in benign lesions such as inflammation, causing false-positive results and misinterpretation for diagnosis [11]. Tremendous efforts have been reported in the literature to deal with this false-positive issue using different tracers e.g. labeled amino acids [12]. However, these tracers have predominantly been used in the research environment with limited clinical usage thus far [13]. In parallel with the introduction of new tracers, researchers also proposed different measuring protocols such a as dual time point imaging procedure and dynamic PET with tracer kinetic modeling [14, 15]. Usually, such modeling procedures are complex, requiring longer scanning sessions, invasive arterial blood sampling, tracer analysis and complex data processing, making the technique less appropriate in daily clinical practice.

Taking the above into account, there is an urgent need to find complementary non-invasive, clinical biomarkers that are able to better discriminate between false positive and true positive results.

In recent years, metabolomics or metabolite profiling/phenotyping, has been used to investigate metabolic changes in plasma associated with lung cancer [16–19]. Metabolomics is the study of substrates and products of metabolism, which are influenced by both genetic and environmental factors. Metabolites and their concentrations directly reflect the underlying biochemical activity of cells and represent the phenotype. Currently, mass spectrometry coupled to different chromatographic separation methods and ^1^H-NMR spectroscopy are the major tools to analyze a large number of metabolites simultaneously. Several research groups have developed a ^1^H-NMR derived metabolic signature of lung cancer in tissue or plasma [16, 17, 19–21]. However, the patient populations in these studies were rather limited.

Recently, our research group was able to detect lung cancer in a population of 269 patients and 347 controls with a sensitivity of 78% and a specificity of 92% by means of the metabolic phenotype of blood plasma [16]. In general, the principal metabolic alterations reported for lung cancer include changes in amino acid metabolism, choline phospholipid metabolism, glycolysis, one-carbon metabolism and lipid metabolism.

Metabolic phenotyping by ^1^H-NMR spectroscopy of patients with benign PET-positive lesions and of patients with lung cancer might result in the discovery of new selective biomarkers with diagnostic potential that can influence the decision-making in case of positive screening results.

The present study is the first in the field of metabolomics that aims to investigate whether the ^1^H-NMR-derived metabolic phenotype of blood plasma allows to discriminate between patients with pulmonary inflammatory disease and lung cancer, as well as between patients with lung inflammation and controls.

## Methods

### Subjects

The presented study is a retrospective analysis of the monocentral NCT02024113-trial [16]. The investigators of the original study evaluated whether the metabolic profile of blood plasma allows to detect lung cancer. Subjects were assigned to three groups: patients with lung cancer, patients with lung inflammation and a control group with similar baseline clinical characteristics. The lung cancer patients (*N* = 269) were included in the Limburg PET Center (Hasselt, Belgium) from March 2011 to June 2014. The diagnosis was confirmed by a biopsy or by interpretation of the images by a respiratory physician specialized in the interpretation of clinical and radiological lung cancer data. The carcinomas were staged according to the 7th edition of the tumor, node, metastasis criteria for lung cancer established by the International Association for the Study of Lung Cancer (IASLC) in 2007 [22]. Patients with initially suspicious CT findings that underwent PET-CT were classified as inflammation after the exclusion of malignant disease by follow-up or a tissue biopsy (*N* = 108). A check of the medical files was accomplished at the time of statistical analysis to confirm the absence of cancer for the 21 (19, 4%) cases of inflammation without a pathologic confirmed diagnosis.

The controls (*N* = 347) were patients with non-cancerous diseases who were referred to the department of Nuclear Medicine (Ziekenhuis Oost-Limburg, Genk) for a stress examination of the heart between March 2012 and June 2014. The absence of malignant disease was confirmed on the basis of the hospital medical files.

The exclusion criteria for all patients (lung cancer – lung inflammation) and controls were as follows: not fasted for at least 6 h, fasting blood glucose ≥200 mg/dl, medication intake on the morning of blood sampling and a treatment or history of cancer in the past 5 years. Characteristics of the subjects included in this study are summarized in Table 1.

**Table 1:** Clinical and pathological characteristics of the study population

	Lung cancer	Inflammation	Controls
Gender			
Female	82 (30.5%)	35 (32.4%)	169 (48.6%)
Male	187 (69.5%)	73 (67.6%)	179 (51.4%)
Age (mean ± SD)	68.1 ± 9.9	63.3 ± 11.5	67.3 ± 11.0
SUV (mean ± SD)	12.1 ± 7.6	4.3 ± 2.8	
Diabetes			
No	222 (82.5%)	98 (90.7%)	280 (80.5%)
Yes	47 (17.5%)	10 (9.3%)	68 (19.5%)
Glycemia (mean ± SD)	105.5 ± 21.3	101.7 ± 20.0	
Smoking habits			
Former	130 (48.3%)	47 (43.5%)	147 (42.2%)
Never	10 (3.7%)	15 (13.9%)	132 (38.0%)
Active	129 (48%)	41 (38%)	69 (19.8%)
Unknown	0 (0%)	5 (4.6%)	0 (0%)
TNM stage			
IA	53 (19.7%)		
IB	22 (8.2%)		
IIA	16 (5.9%)		
IIB	16 (5.9%)		
IIIA	63 (23.4%)		
IIIB	28 (10.5%)		
IV	71 (26.4%)		
Histology			
CARCINOMA			
Adenocarcinoma	101 (37.5%)		
Adenosquamous	5 (1.9%)		
Squamous	71 (26.4%)		
NOS	9 (3.3%)		
Carcinoid	5 (1.9%)		
SCLC	38 (14.1%)		
No histology	35 (13%)		
Other	5 (1.9%)		
INFLAMMATION			
Pneumonia	54 (50.0%)		
Sarcoidosis	6 (5.6%)		
Granuloma^a^	6 (5.6%)		
Mycobacteria	5 (4.6%)		
Antracosilicosis	9 (8.3%)		
Unknown	21 (19.4%)		
Miscellaneous	7 (6.5%)		

*NOS* = not otherwise specified, *SCLC* small cell lung carcinoma, *SD* standard deviation, *TNM* tumor-node-metastasis; ^a^other than sarcoidosis

### Blood sampling, sample preparation and NMR analysis

10 cc venous blood (10 cc), of fasting patients, was collected in lithium-heparin tubes and stored within 5 min at 4 °C. Samples were centrifuged at 1600 *g* for 15 min, within 8 h after collection. Plasma aliquots (500 μl) were transferred into cryovals and stored at − 80 °C. After thawing, the aliquots were centrifuged at 13000 *g* for 4 min at 4 °C. Subsequently, 200 μl of the supernatant was diluted with 600 μl deuterium oxide (D_2_O) that contained 0.3 μg/μl trimethylsilyl-2,2,3,3-tetradeuteropropionic acid (TSP) as chemical shift reference. Until ^1^H-NMR analysis, the prepared samples were placed on ice. Samples were mixed and transferred into NMR sample tubes (5 mm) and were acclimatized to 21.2 °C during 7 min. All ^1^H-NMR spectra were recorded with an Inova 400 MHz spectrometer (Agilent Technologies Inc.) at 21.2 °C. A transverse relaxation (T2-weighted) edited Carr-Purcell-Meiboom-Gill sequence (total spin-echo time: 32 ms; interpulse delay: 0.1 ms) was acquired. This was preceded by an initial preparation delay of 0.5 s and 3 s presaturation for water suppression. Other acquisition parameters were: spectral width 6000 Hz; acquisition time 1.1 s, 13 k data points and 96 scans. Before Fourier-transformation, each free induction decay was zero-filled to 65 k points, multiplied by a line broadening of 0.7 Hz, phased and, referenced to TSP. By spiking the plasma of a healthy volunteer with known metabolites (for each metabolite, a different sample with plasma from the plasma pool), the NMR spectrum was segmented into 110 fixed integration regions (IRs) [23]. Water (4.7–5.2 ppm) and TSP (− 0.3–0.3 ppm) resonances were excluded. These spiking experiments allowed us to identify the metabolites of 87 IRs. The remaining 23 IRs originate from non-identified substances and broad lipid signals. Subsequently, the spectra were baseline corrected and integrated. The metabolic profile consists of 110 numerical integration values, i.e. the area under the peaks of these 110 integration regions, representing the metabolite concentrations. By normalizing the integration values to the total integrated area, except water and TSP, relative concentrations were obtained. These are the variables for the statistical PLS-LDA multivariate analysis. The spiking methodology was preferred above peak alignments based on chemical shift values reported for different matrices and even non-human species [24–26]. In addition, in contrast with binning, the spiking method avoids the splitting of peaks into parts which may result in a loss of discriminating power. These issues were the rationale for using the spiking method.

### Positron emission tomography/computed tomography (PET-CT)

Static PET-CT (GEMINI TF Big Bore, Philips) images were acquired and assessed retrospectively with commercially available software (Hermes Medical Solutions, Hermes Hybrid Viewer) to measure the SUV_max_. PET-CT was performed after at least 6 hours in the fasting state and 1 hour after the administration of 3.75 MBq/kg ^18^F-FDG. Patients with serum glucose levels ≥200 mg/dl were excluded. First, the imaging field was determined by a scout scan. Thereafter, a low dose CT of 30 s (mAs: 80–175; kV: 120; slice thickness: 5 mm), which ranged from the mid thighs to the base of the skull, was performed. The CT images were reconstructed on a 512–512 matrix. Next, a PET-scan of 15–20 min was performed. Depending on the body mass index (BMI) of the patient, the emission time per bed position ranged from 1 to 2 min.

### Statistical analysis

Sensitivity, specificity, positive and negative predictive values (PPV,NPV) and misclassification error (MCE) were calculated in all the patients that underwent PET-CT (*N* = 377).

In order to detect significant differences between the expression levels of metabolites in controls and patients with lung inflammation, and between patients with lung inflammation and lung cancer, a univariate t-test analysis with a correction for multiple testing by Benjamini and Hochberg was performed using the free R (2.15.0) software package [27]. For all IRs, the results of the t-test (*p*-values) and the magnitude of the differences between the two groups were combined in a volcano plot to present a visual overview of the most meaningful differences (Additional file 1: Figure S1). To evaluate the potential diagnostic performance of IR variables that were significantly different between the groups, receiver operating characteristic curves (ROC) were calculated. In addition, classification of the disease status (cancer/inflammation and control/inflammation) was conducted using the partial least square-linear discriminant analysis (PLS-LDA) method in which the least absolute shrinkage and selection operator (LASSO) method was used for top K feature selection (with K, the number of IRs to be included in the classifier model). LASSO is a method that is often used for modeling high dimensional data when the number of possible predictors is relatively high [28]. The LASSO procedure is used to select top K variables in predictive models and has the advantage that it penalizes for the number of predictors in the model, i.e. the LASSO method selects the minimal set of predictors that lead to the best prediction [29]. In this study, the LASSO method was used as a variable selection method to select the integration regions that best distinguish between cancer/inflammation, i.e. to select the top K best IRs for inclusion in the classifier signature. After selecting the top-K list, the PLS-LDA method was used for the classification using a four-fold cross validation procedure**.** To evaluate the multivariate approach, PLS-LDA classifier models with different top K signatures are constructed and compared with respect to their diagnostic characteristics.

**Fig. 1:**
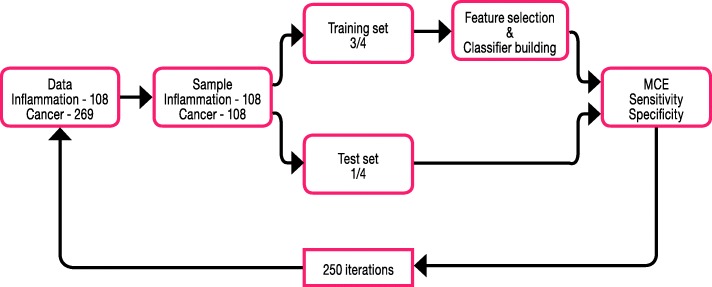
Classification workflow to differentiate between lung inflammation and lung cancer. MCE = misclassification error

PLS is a latent variable regression method that maximizes the covariance between the predictors X (metabolic data) and the response Y (disease). A discriminant variant of PLS, PLS-LDA, refers to a classification method in which each observation is described by one out of two (or more) categories [17, 30, 31]. In the unbalanced population of 29% (*N* = 108) inflammation patients and 71% (*N* = 269) cancer patients, classification procedures will typically lead to biased results as the procedures have the tendency to classify inflammation as cancer. To overcome this problem, we sampled 108 cancer patients ad random out of 269 lung cancer patients and thus develop the classifier on a balanced dataset. A similar approach was used between lung inflammation and controls (random selection of 108 out of 347). This random selection of cancer patients is applied in a loop of a 250 four-fold cross validation which implies that the classifier is evaluated 1000 times for 250 random selections of 108 cancer patients. This step is needed since it is unwanted that one specific random selection of 108 patients will determine the results. Once a subset of 108 cancer patients was randomly selected, the four-fold cross-validation procedure was as follows: a training set (3/4 of the subjects) was used for feature selection and classification and a validation test set (1/4 of the subjects) was used for independent internal validation [32]. The test-set was used to validate the classification ability of the trained models, generating a mean misclassification error (MCE) and a mean sensitivity and specificity. The same approach (Fig. 1) was used for feature selection and classification of the inflammation-control dataset and the lung cancer-control dataset. In order to evaluate the performance of the classifier across the different cancer stages and whether specific cancer stages have a tendency to be misclassified, a leave-one-out-cross-validation (LOOCV) was applied (see Additional file 2 for a description of the LOOCV method).

In addition to classification models based solely on the metabolite NMR data, models including in addition the SUV_max_ parameter obtained from metabolic PET-CT imaging are evaluated as well.

## Results

### Diagnostic characteristics of PET-CT

Sensitivity, specificity, PPV and NPV of PET-CT for diagnosis of lung cancer (based on a widely accepted clinical value of SUV_max_ ≥ 2.5) were 96, 23, 76 and 71%, respectively.

### ^1^H-NMR signature of lung inflammation versus lung cancer

In a ^1^H-NMR spectrum, hydrogen atoms with different chemical environments give rise to signals at different positions (i.e. at different chemical shifts, expressed in ppm) in the spectrum. Since most metabolites have hydrogen atoms with different chemical environments in their chemical structure, they will give rise to more than one signal in the ^1^H-NMR spectrum (Additional file 3: Figure S2). This explains why i) the NMR spectra are segmented in 110 regions on the basis of published results describing the spiking of a reference plasma pool with known metabolites and ii) these 110 regions represent less than 110 metabolites [23]. It further explains why some regions in the spectrum do represent a single metabolite, while other regions consist of overlapping signals of several metabolites. These 110 regions are integrated (the area under the peaks is a measure for the concentration of the constituent metabolites) and normalized relative to the total integrated area (except this of water and TSP), resulting in 110 numerical values that represent the relative metabolite concentrations and form the metabolic signature, and which are referred to as variables IR1,…IR110 in the statistics.

**Fig. 2:**
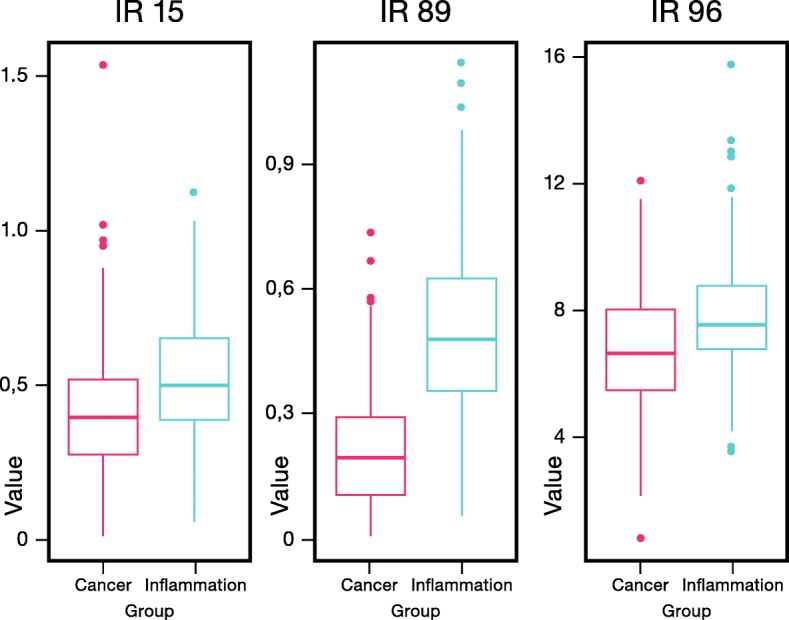
Box-plots of IR15, IR89 and IR96 reveal significant differences between patients with lung inflammation and lung cancer patients. Despite the relatively small fold change of IR89 (Additional file 1: Figure S1), the integration value (and so relative glutamate concentration) is significantly higher in the inflammation group. IR = integration region. IR89 represents glutamate and methionine, IR15 represent tyrosine and IR96 contains signals from alanine, isoleucine and lysine. IR = integration region

**Fig. 3:**
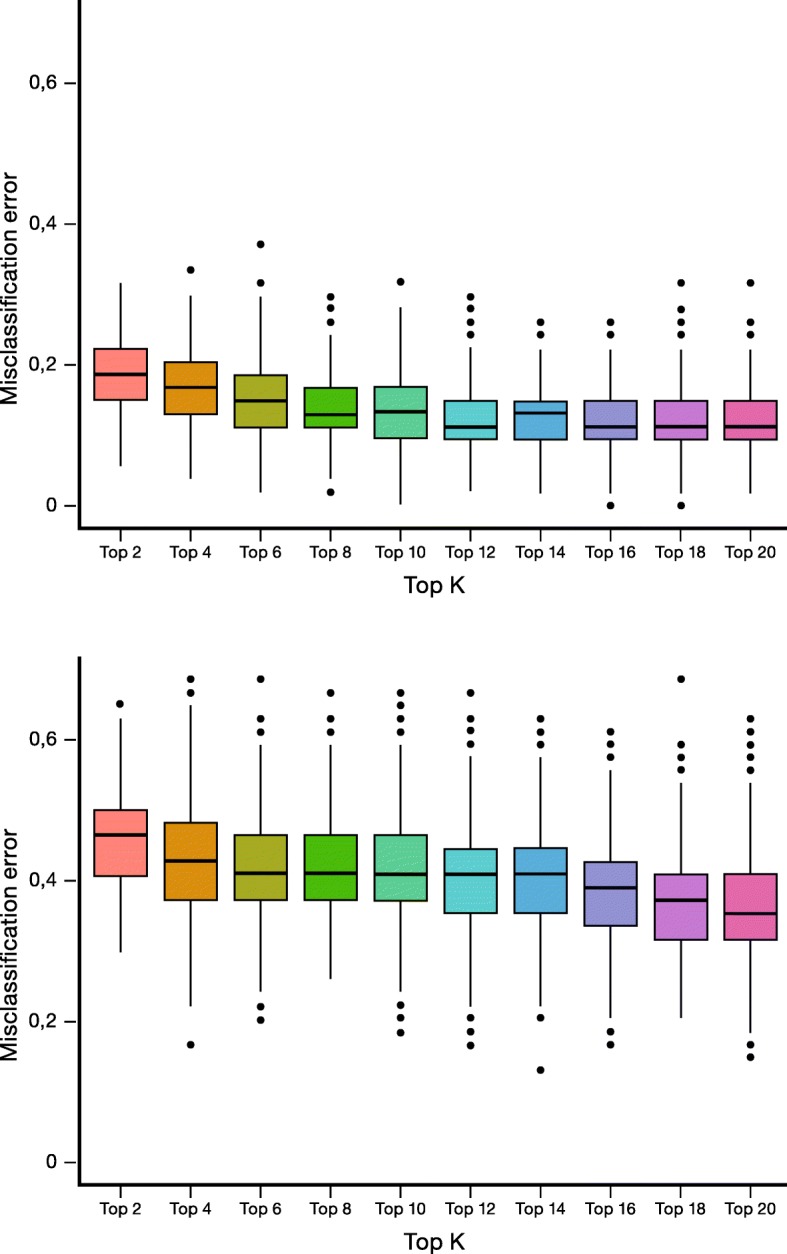
MCE as a function of top K feature selection for the full data set (top) and after withdrawal of IR89 from the data set (bottom) reveals a strong increase in MCE between patients with lung inflammation and lung cancer upon removal of IR89. IR = integration region, MCE = misclassification error

Univariate statistical analysis indicates that IRs 15, 89 and 96 are the most significant variables in the differentiation between lung cancer patients and patients with lung inflammation (Additional file 1: Figure S1). These IRs reflect the relative plasma concentrations of respectively tyrosine (IR15), glutamate and methionine (IR89) and of a group consisting of alanine, isoleucine and lysine (IR96). Plotting the value of IR89 reveals a clear and significant difference between lung cancer patients and patients with lung inflammation (Fig. 2). In addition, IR89 was selected in all the cross-validation runs of the multivariate PLS-LDA statistics by the LASSO top K feature selection procedure. As the main goal of this study concerns the discrimination between patients with lung cancer and lung inflammation, the whole signature might be of interest, but to avoid overfitting of the current data matrix, a LASSO approach was introduced to select the top K most important (differentiating) variables. From the Additional file 4: Table S1, it can be seen that the MCE, sensitivity and specificity of the PLS-LDA model do not further improve if the signature size exceeds the top 16 IRs. The classification model constructed with the top 16 variables results in an average MCE of 12% (Fig. 3, top), a sensitivity of 89% and a specificity of 87%. The performance of models using a smaller top K feature selection is also demonstrated in Additional file 4: Table S1 and shows that, for the current data matrix, the model performance becomes worse if less than the top 16 variables are used. The increase of MCE and decrease of the sensitivity and specificity in models using less top K features indicate that a minimal set of variables remains essential for an optimal differentiation. As IR89 was selected in all the LASSO selections, its importance was further examined by its removal from the data, resulting in an increase of the MCE from 12% to 38% (Fig. 3, bottom) and a drop in sensitivity and specificity from 89% to 62% and from 87% to 62%, respectively. This large increase in MCE demonstrates that IR89 strongly drives the classification. IR89 is assigned to the most downfield part of the multiplet of the β-CH_2_ protons of glutamate, situated between 2.197 and 2.218 ppm, as proven by spiking experiments [23]. It was further demonstrated that this region might only contain additional signals of the β-CH_2_ protons of methionine (Additional file 5: Figure S3). The presence of signals of other metabolites can be excluded via spiking with other metabolites, including all amino acids. However, the spiking experiments also have shown that IR72 only comprises the triplet signal of the γ-CH_2_ protons of methionine between 2.63 and 2.66 ppm. Since this IR72 is not increased in case of inflammation, we assign the increase of IR89 in case of inflammation to an increase in glutamate. Mean relative serum levels of glutamate are 0.159 (SD 0.156) in cancer; 0.485 (SD 0.237) in inflammation and 0.152 (SD 0.113) in controls.

Incorporation of the SUV_max_ value, obtained by PET-CT, as an additional variable in the PLS-LDA model results in only modest improvements, a MCE of 10% and a sensitivity and specificity of 89% and 91%, respectively.

**Fig. 4:**
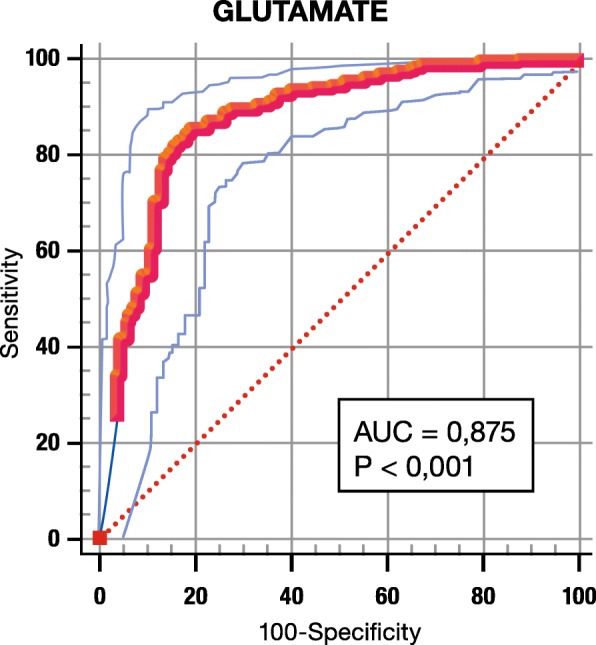
ROC curve for glutamate. A low glutamate concentration is considered as diagnostic for cancer. The cut-off point with the highest sensitivity and lowest 100-specificity is 0, 31. *p* value < 0,001, area under the curve (AUC) 0,875

**Fig. 5:**
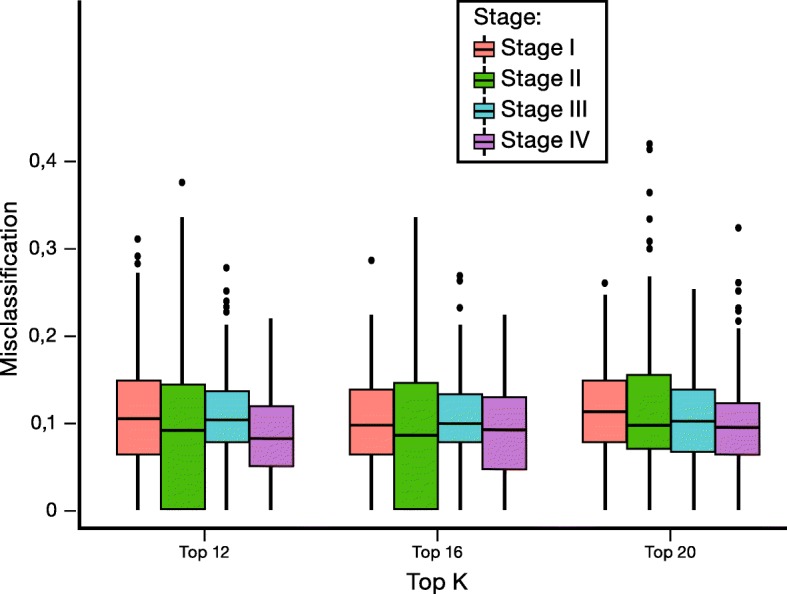
Boxplots of MCE for different cancer stages reveal that stage does not influence classification

To examine the potential role of the relative glutamate concentration as a single diagnostic marker to differentiate between lung cancer and lung inflammation, we constructed a receiver operating characteristic (ROC) curve. As demonstrated in Fig. 4, multiple cut points are possible to classify the patient within the lung inflammation or lung cancer group. Taken that the test is considered positive for cancer in case of low glutamate concentrations, the optimal cut-off point (highest sensitivity and 1-specificity) for cancer diagnosis corresponds to a relative glutamate level of ≤0.31 (AUC of 0.88). The combination of the highest sensitivity and 1-specificity was obtained at the cut-off point of a relative glutamate concentration of 0.31. This cut-off value corresponds to a sensitivity of 85%, a specificity of 81%, and an AUC of 0.88 (*p* value < 0.0001). The PPV and NPV are 92 and 69%, respectively. Assuming that PET-positive lesions have an SUV_max_ ≥ 2.5, a low relative glutamate concentration results in the diagnosis of lung cancer with a sensitivity of 100% and with a very high PPV of 94%. In PET-negative patients, a high relative glutamate concentration excludes lung cancer in all patients (NPV 100%). In cases of contradictory results i.e. SUV_max_ ≥ 2.5 and relative glutamate level > 0.31 or SUV_max_ < 2.5 and relative glutamate level ≤ 0.31, 19% of the cancer diagnoses are missed. In order to investigate the performance of the classifier across cancer stages and whether specific cancer stages have a tendency to be misclassified, an additional analyses was conducted in which the MCE per cancer stage was calculated. The classification was done using the leave-one-out-cross-validation (LOOCV) method of which an elaborate explanation can be found in the Additional file 2 of the paper. The table in the Additional file 2 shows the results obtained for the overall MCE, sensitivity and specificity. As shown in the boxplots of Fig. 5 and Table 2, the MCE per cancer stage indicates that the performance of the classifier is similar across stages. Relative glutamate levels do not significantly differ between lung cancer stages (*p* value = 0.3): stage I 0.161 (SD 0.159), stage II 0.115 (SD 0.112), stage III 0.177 (SD 0.165) and stage IV 0.155 (SD 0.156).

**Table 2:** MCE (%) results of the leave-one-out-cross-validation (LOOCV) for different top K signature sizes per cancer stage

Top K	Overall stage (%)	Stage I (%)	Stage II (%)	Stage III (%)	Stage IV (%)
12	11	11	10	11	9
16	10	10	10	10	10
20	10	11	10	10	9

*LOOCV* leave-one-out cross validation, *MCE* misclassification error

### ^1^H-NMR signature of lung inflammation versus control

Also here, univariate analysis indicates glutamate as the most significant variable to differentiate between patients with lung inflammation and controls. Relative glutamate concentrations are significantly higher in patients with inflammation than in controls (*p* value < 0.0001): 0.485 (SD 0.237) versus 0.152 (SD 0.113).

In addition, glutamate is selected in the top 16 of all cross-validation runs by the LASSO feature selection method. The PLS-LDA classification models result in an average MCE of 7%, a sensitivity of 92% and a specificity of 94%. Classification after removing IR89 from the top 16 selection list resulted in much weaker PLS-LDA models showing an increase of the average MCE from 7% to 29% and a decrease in sensitivity and specificity from 92% to 75% and 94% to 75%, respectively.

### ^1^H-NMR signature of lung cancer versus control

Here, glutamate clearly becomes less important as it was selected in only 58% of the 1000 cross-validation runs by the LASSO feature selection method. The PLS-LDA model results in a MCE of 25%, a sensitivity of 68% and a specificity of 82%. A substantial number of constructed classification models (42%) did not include glutamate, indicating that it is not very important in the differentiation between lung cancer patients and controls. The relative concentration of glutamate did not significantly differ between lung cancer patients and controls (*p* value = 1): 0.159 (SD 0.156) versus 0.152 (SD 0.113).

## Discussion

In the United States, regular low-dose CT screening has been recommended for smokers and ex-smokers at high risk of developing lung cancer [5]. However, the main challenge for lung cancer screening by CT remains the high prevalence of pulmonary nodules and/or lymph nodes, and a relatively low incidence of lung cancer in the screened population [4, 33, 34]. This results in a low PPV after exclusion of lung cancer by additional imaging and potential harmful procedures, such as tissue biopsies. The aim of this study is to search for metabolites that discriminate between lung cancer patients and patients with lung inflammation by means of the plasma metabolic fingerprint. The metabolic phenotype or fingerprint consists of a large number of variables, each of them representing a single or several metabolite concentrations. To the best of our knowledge, this study is the first in the field of metabolomics that investigates the metabolic differences in blood plasma of patients with lung inflammation and lung cancer.

This study indicates that the metabolic phenotype of blood plasma, and particularly the region representing glutamate, allows to discriminate between patients with lung inflammation and with lung cancer, as well as between patients with lung inflammation and controls. These results strongly suggest the role of glutamate as a selective inflammatory marker in lung diseases. Ideally, after detection of a suspicious lesion on chest-CT, differences in the plasma metabolic profile in combination with PET findings may add valuable information about the underlying disease, i.e. cancer versus inflammation. This approach may reduce the need of invasive diagnostic procedures when the lesion has inflammatory characteristics.

Analytical approaches, such as ^1^H-NMR spectroscopy, generate a large number of variables per sample, resulting in models with a risk of overfitting. A careful selection of the appropriate statistical method is necessary as each of the techniques has advantages and disadvantages. The choice of method is dependent on the type of data: missing values, influence of outliers, predictive power, etc. [30]. In the field of metabolomics, there is an increasing interest in PLS-LDA since it reduces the dimensionality of the spectroscopic data and can handle the noisy and collinear data from the experiment. Moreover, it is available in most of the statistical software packages.

Glutamate may have a key role in the differentiation between lung inflammation and lung cancer. Univariate t-test analysis with correction for multiple testing, shows that the glutamate concentration, represented by IR89, is the most significant variable with the smallest *p*-value and a signal intensity which is significantly higher for lung inflammation as compared to cancer (Fig. 2 and Additional file 3: Figure S2). The differentiating power of this variable is stressed by multivariate PLS-LDA statistics showing an increase of the MCE with 26% (from 12% to 38%) after removing it from the dataset.

Addition of the SUV_max_ parameter, obtained by PET-CT, to the dataset has only a modest influence on the classification (e.g. a decrease of the MCE from 12% to 10%), indicating that the SUV_max_ has no significant power to differentiate between lung inflammation and lung cancer. This is supported by the limited specificity of PET-CT in excluding malignancy on the basis of the SUV_max_ value and the consensus that a metabolically active lesion requires histological assessment [7].

MCE, sensitivities and specificities have the tendency to stabilize when the metabolic signature contains 16 variables. This means that despite the importance of glutamate, other IRs may have additional value in the classification process. Glutamate, however, was selected in all the LASSO models and was the most significant variable in the univariate analyses. Therefore, the diagnostic potential of glutamate as a single marker was further evaluated by a ROC curve.

To diagnose lung cancer, and in comparison with PET-CT itself, a relative glutamate level ≤ 0.31 has a lower sensitivity (85% versus 96%), a significant higher specificity (81% versus 23%), a higher PPV (92% versus 76%) and a comparable NPV (69% versus 71%). Due to this lower sensitivity (i.e. more false negative results) and the resulting NPV, glutamate as a single marker is insufficient to exclude lung cancer. To overcome these limitations, we propose to measure plasma glutamate in complement to PET-CT. In patients with both PET-positive lesions and low relative glutamate levels (suggestive for cancer), this procedure leads to a sensitivity and PPV to diagnose lung cancer of 100% (no false negatives) and 96% (higher true positive results than for PET/CT alone), respectively. In this patient group, a tissue biopsy or resection is indispensable to obtain the histology and to guide further therapy. A negative PET-CT and a high relative glutamate concentration (suggestive for inflammation) excludes lung cancer with a NPV of 100%. Here, further follow-up with CT but without invasive procedures seems to be justified. Caution is needed in patients with conflictive results, i.e. PET-positive patients with a high glutamate concentration or PET-negative patients with a low relative glutamate concentration. In these patients a tissue biopsy or more intensive follow-up is needed to exclude or confirm the presence of lung cancer since 19% of lung cancers remain undetected in this group.

As undetermined imaging results are less frequent in more advanced disease stages than in early stages, we compared the mean relative glutamate concentration in different stages by the leave-one-out-cross-validation (LOOCV) method. No significant differences were found between the glutamate levels of early (I and II), locally advanced (III) and advanced stages (IV), as demonstrated in Fig. 5 and Additional file 2.

To confirm the potential value of glutamate as a marker for lung inflammation, a PLS-LDA analysis was performed to discriminate between patients with lung inflammation and controls. The resulting model has a very small MCE of 7% and a high sensitivity and specificity. Relative glutamate concentrations were significantly higher in patients with lung inflammation compared to controls, supporting the importance of glutamate as an inflammatory biomarker. Building a ROC curve to determine an optimal cut-off in a diagnostic test for lung inflammation seems less relevant as common markers as C-reactive protein, sedimentation rate and leukocytosis are robust biomarkers.

Unfortunately, due to the retrospective nature of this study, these parameters were not available at the moment of the ^1^H-NMR analysis, preventing to look for possible correlations between the glutamate concentration and these markers.

Glutamate is a non-essential amino acid that accounts for 15% of the total amino acids in dietary proteins. Since the blood samples in this study were taken after an overnight fast and glutamate concentrations are normalized within 105 min after ingestion, the influence of glutamate intake should be negligible [35]. Dysregulation of the glutamine-glutamate metabolism is reported for cancer cells [36]. Cancer cells use glutamine as a source of carbon for further anabolic pathways (oxidation) and glutamine is hereto transported into the cells by the alanine-serine-cysteine-transporter-2. As a nitrogen donor for the synthesis of DNA and RNA building blocks, glutamine is converted into glutamate [37, 38]. However, glutamine can also be exported out of the cell by antiporters in exchange for other non-essential amino acids through the L-type amino-acid transporter [39]. Glutamine-derived glutamate also fulfills the role of a primary nitrogen donor for the synthesis of non-essential amino acids and is a precursor of the major cellular antioxidant glutathione (GSH) [40, 41]. Increased GSH synthesis has been demonstrated in lung cancer tissue by Blair et al. [42]. Higher levels of GSH have been related to apoptosis resistance [43]. Glutamate that is not incorporated into GSH or involved in the synthesis of amino acids is converted to α-ketoglutarate (α-KG) through oxidative deamination. By this reaction, the glutamine-derived α-KG is utilized to replenish synthetic intermediates of the Krebs cycle, a phenomenon known as anaplerosis. Instead of the complete oxidation of glutamine to ATP, the mitochondria of cancer cells shunt glutamine into citrate for the production of NADPH and lipid synthesis, and into malate which can be converted into pyruvate and NADPH [36]. The need of glutamate in the synthesis of GSH and macromolecules such as lipids and polynucleotides, may explain the lower levels of glutamate in the plasma of cancer patients compared to patients with lung inflammation. During inflammation the increase of vascular permeability facilitates the uptake of glutamate in the inflamed tissues. As part of the immune response generated by inflammation, cytotoxic T-cells are able to induce apoptosis in the inflamed tissue, thereby releasing intracellular glutamate. This process may explain the higher glutamate plasma concentration in patients with lung inflammation.

Regarding the role of glutamate in discriminating lung cancer patients from controls, the relative glutamate concentrations are not significantly different. As a marker of lung inflammation, glutamate is not able to distinguish between cancer patients and controls. Recently, our research group has demonstrated that the metabolic phenotype of blood plasma enables to distinguish lung cancer patients from controls [16]. The fact that glutamate did not appear in the list of discriminating variables confirms our results and interpretation.

The generalizability of the results is subject to certain limitations. First, due to the retrospective nature of the study, other markers for inflammation such as C-reactive protein, sedimentation rate and leukocytosis were not available at the time of inclusion. Additionally, uncontrolled factors such as co-morbidities and their treatments might be possible confounders. It goes without saying that the role of glutamate as a potential marker of lung inflammation needs further evaluation in a prospective study with external validation and attention for possible confounders. Also the potential role of glutamate as a single biomarker for lung inflammation in a targeted approach needs to be further explored by another analytical technique such as HPLC-MS. And finally, the correlation with other markers for inflammation needs further investigation.

## Conclusion

The aim of this study is to investigate whether the ^1^H-NMR-derived metabolic phenotype of blood plasma allows to discriminate between patients with lung inflammation and lung cancer. To the best of our knowledge, the presented study is the first to investigate differences in the metabolic composition of blood plasma between patients with lung inflammation and lung cancer. The glutamate concentration is found to be the most important metabolite in the discrimination. Using a relative glutamate level ≤ 0.31 as a single criterion results in a lower sensitivity than PET-CT itself but also in a higher specificity of 81%. Using the combination of two criteria, i.e. a SUV_max_ ≥ 2.5 and a relative glutamate level ≤ 0.31 is likely to correspond with the diagnosis of lung cancer and immediate referral to a respiratory physician is mandatory. In contrast, a SUV_max_ < 2.5 and a relative glutamate level > 0.31 is rather suggestive for lung inflammation and a wait-and-see attitude seems justified. Caution is needed for patients with conflicting results between the SUV_max_ value and the relative glutamate concentration. In these patients a tissue biopsy or more intensive follow-up is needed to exclude or confirm the presence of lung cancer since 19% of the lung cancers remain undetected in this group. Although lung cancer screening studies are compromised by a low PPV, a subsequent combination of PET-positive lesions and low glutamate concentration has a PPV of 94%, implicating that less patients with a positive PET-CT may be exposed to unnecessary invasive diagnostic procedures. However, before possible clinical implementation, a larger prospective study with external validation is obligatory and the potential of glutamate as a single biomarker for lung inflammation needs to be confirmed by another analytical technique such as HPLC-MS.

## Additional files

Additional file 1: **Figure S1.** Volcano plot presenting an overview of the most meaningful differences between the metabolic fingerprints of lung cancer and lung inflammation. The plot displays fold change (X-axis) versus the absolute value of the log *p*-value (Y-axis). The blue dots represent variables with significant *p*-values. The green dots represent variables with a high fold change, but non-significant *p*-value. The red dots represent variables with a negligible fold change and non-significant *p*-value. IR = integration region. (PDF 525 kb)

Additional file 2: Elaborate explanation of the Leave-One-Out-Cross-Validation (COOCV) method and evaluation of the classifier across cancer stages. (DOCX 22 kb)

Additional file 3: **Figure S2.** Focus on the ^1^H-NMR regions IR89, IR90 and IR91. The β-CH_2_ protons of glutamate are diastereotopic since they are located on a carbon atom next to an asymmetric carbon atom. This results in a complex multiplet of several peaks situated between 2.03 and 2.22 ppm, and appearing in the following three integration regions: IR89 = glutamate and methionine; IR90 = glutamate, glutamine, proline and methionine; and IR91 = C**H**_2_-C=O or C**H**_2_-CH=CH- of fatty acids, glutamate, isoleucine, methionine and proline. A: ^1^H-NMR spectrum of a lung cancer patient. B:: ^1^H-NMR spectrum of a patient with lung inflammation. (PDF 512 kb)

Additional file 4: **Table S1.** Performance (mean) of the PLS-LDA classification for different top K signature sizes, full data. (DOCX 14 kb)

Additional file 5: **Figure S3.** Spiking experiments glutamate and methionine. These experiments demonstrate that the proton signal of IR89 is assigned to the most downfield part (left side) of the multiplet of the β-CH_2_ protons of glutamate. However, this region might also contain signals of the β-CH_2_ protons of methionine (right side). A: NMR spectrum of the plasma of a healthy person after spiking with Glu (left) and Met (right). B: NMR spectrum of a healthy person. (PDF 540 kb)

## Abbreviations

^18^F-FDG: ^18^F-fluorodeoxyglucose
^1^H-NMR: Proton Nuclear Magnetic Resonance
CT: Computed Tomography
GSH: Glutathione
HPLC-MS: High performance liquid chromatography with mass spectrometry
IR: Integration Region
LASSO: Least Absolute Shrinkage and Selection Operator
LOOCV: Leave-one-out cross validation
MCE: misclassification error
NADPH: Reduced Nicotinamide adenine dinucleotide phosphate
NPV: Negative predictive value
PET: Positron Emission Tomography
PET-CT: Positron Emission Tomography – Computed Tomography
PLS-LDA: Partial Least Squares – Linear Discriminant Analysis
ppm: Parts per million
PPV: Positive predictive value
ROC: Receiver operating characteristic curve
SD: Standard deviation
SUV: Standardized Uptake Value
SUV_max_: Maximal Standardized Uptake Value
α –KG: α-Ketoglutarate
