# Supplementary figures and images for: The plasma glutamate concentration as a complementary tool to differentiate benign PET-positive lung lesions from lung cancer
Source: BMC Cancer. 2018 Sep 3;18:868. doi: 10.1186/s12885-018-4755-1 (PMC6122613; doi:10.1186/s12885-018-4755-1)

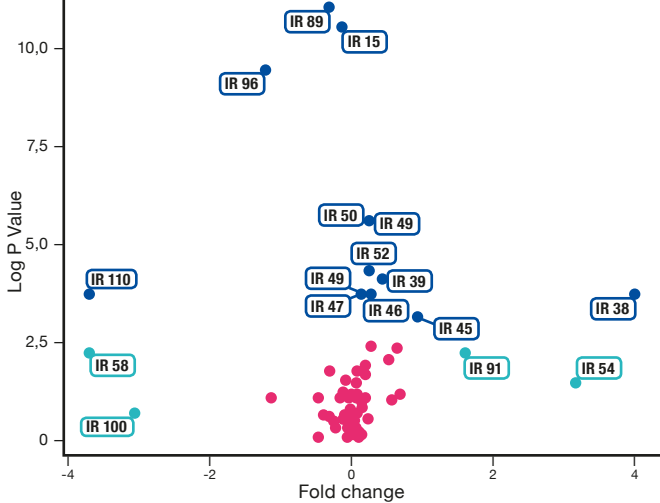

Supplement: Supplementary file 1 — Figure S1. Volcano plot presenting an overview of the most meaningful differences between the metabolic fingerprints of lung cancer and lung inflammation. The plot displays fold change (X-axis) versus the absolute value of the log p-value (Y-axis). The blue dots represent variables with significant p-values. The green dots represent variables with a high fold change, but non-significant p-value. The red dots represent variables with a negligible fold change and non-significant p-value. IR = integration region. (PDF 525 kb) [file 12885_2018_4755_MOESM1_ESM.pdf]

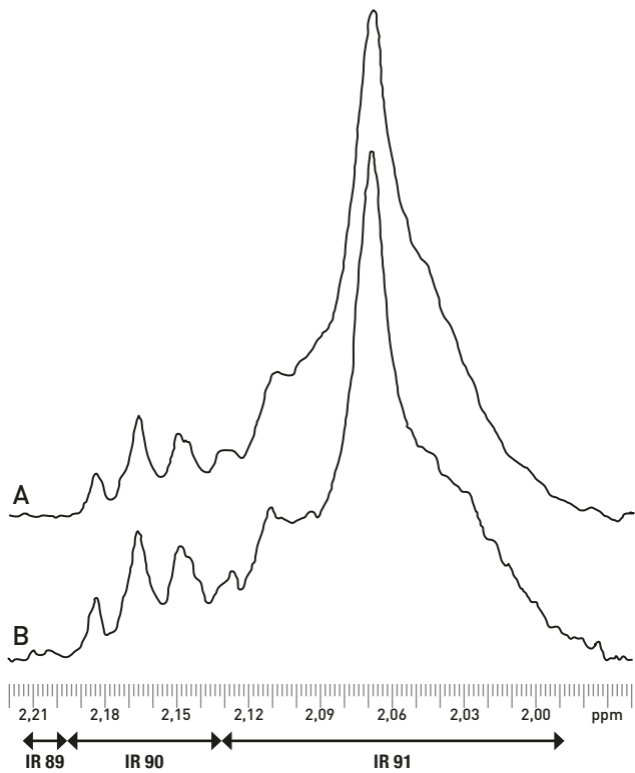

Supplement: Supplementary file 3 — Figure S2. Focus on the 1H-NMR regions IR89, IR90 and IR91. The β-CH2 protons of glutamate are diastereotopic since they are located on a carbon atom next to an asymmetric carbon atom. This results in a complex multiplet of several peaks situated between 2.03 and 2.22 ppm, and appearing in the following three integration regions: IR89 = glutamate and methionine; IR90 = glutamate, glutamine, proline and methionine; and IR91 = CH2-C=O or CH2-CH=CH- of fatty acids, glutamate, isoleucine, methionine and proline. A: 1H-NMR spectrum of a lung cancer patient. B:: 1H-NMR spectrum of a patient with lung inflammation. (PDF 512 kb) [file 12885_2018_4755_MOESM3_ESM.pdf]

**GLUTAMATE SPIKING  
EXPERIMENT**

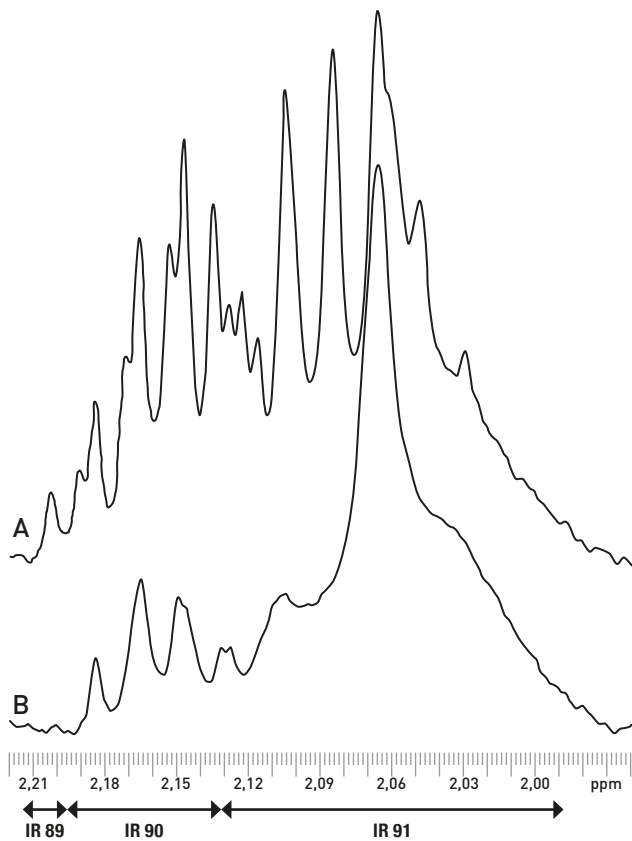

**METHIONINE SPIKING  
EXPERIMENT**

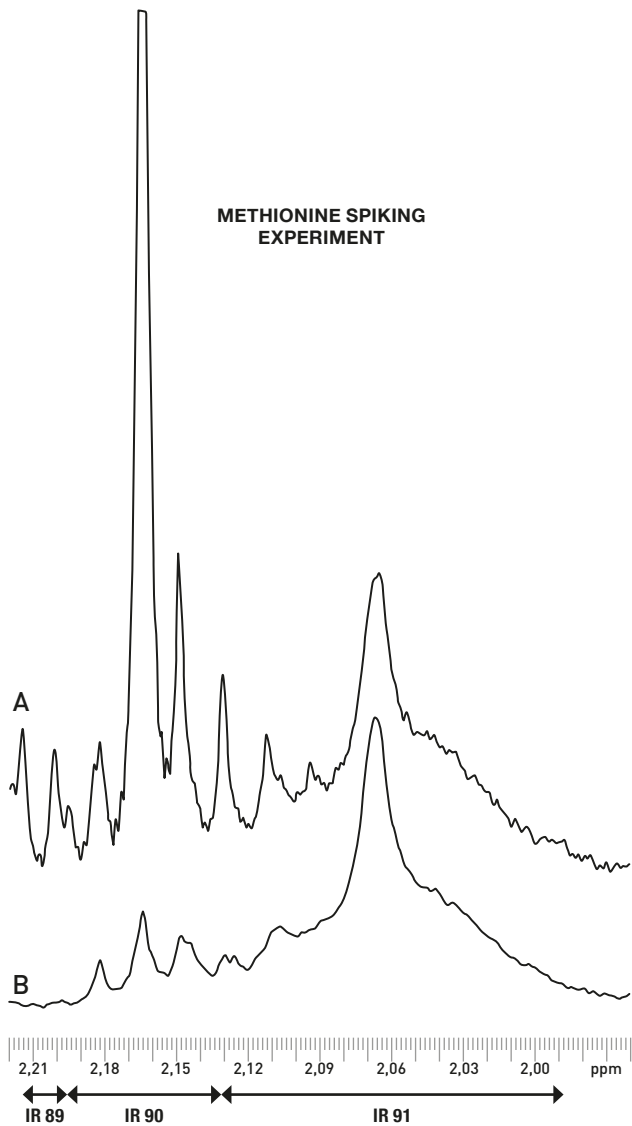

Supplement: Supplementary file 5 — Figure S3. Spiking experiments glutamate and methionine. These experiments demonstrate that the proton signal of IR89 is assigned to the most downfield part (left side) of the multiplet of the β-CH2 protons of glutamate. However, this region might also contain signals of the β-CH2 protons of methionine (right side). A: NMR spectrum of the plasma of a healthy person after spiking with Glu (left) and Met (right). B: NMR spectrum of a healthy person. (PDF 540 kb) [file 12885_2018_4755_MOESM5_ESM.pdf]
