# Supplementary material for: The plasma glutamate concentration as a complementary tool to differentiate benign PET-positive lung lesions from lung cancer
Source: BMC Cancer. 2018 Sep 3;18:868. doi: 10.1186/s12885-018-4755-1 (PMC6122613; doi:10.1186/s12885-018-4755-1)
Supplement: Supplementary file 4 — Table S1. Performance (mean) of the PLS-LDA classification for different top K signature sizes, full data. (DOCX 14 kb) [file 12885_2018_4755_MOESM4_ESM.docx]

**SUPPLEMENTARY TABLES**

Supplementary Table S1 : performance (mean) of the PLS-LDA classification for different top K signature sizes, full data.

|  | Misclassification | Sensitivity | Specificity |
| --- | --- | --- | --- |
| Top 2 | 18 | 85 | 78 |
| Top 4 | 17 | 87 | 80 |
| Top 6 | 15 | 88 | 82 |
| Top 8 | 14 | 88 | 84 |
| Top 10 | 13 | 89 | 85 |
| Top 12 | 12 | 89 | 86 |
| Top 14 | 12 | 89 | 86 |
| Top 16 | 12 | 89 | 87 |
| Top 18 | 12 | 89 | 87 |
| Top 20 | 12 | 89 | 87 |
