# Supplementary material for: The plasma glutamate concentration as a complementary tool to differentiate benign PET-positive lung lesions from lung cancer
Source: BMC Cancer. 2018 Sep 3;18:868. doi: 10.1186/s12885-018-4755-1 (PMC6122613; doi:10.1186/s12885-018-4755-1)
Supplement: Supplementary file 2 — Elaborate explanation of the Leave-One-Out-Cross-Validation (COOCV) method and evaluation of the classifier across cancer stages. (DOCX 22 kb) [file 12885_2018_4755_MOESM2_ESM.docx]

**SUPPLEMENTARY APPENDIX**

**Evaluation of the classifier by means of the Leave-One-Out-Cross-Validation method (LOOCV)A point-by-point response letter must accompany your revised manuscript. This letter must provide a detailed response to each reviewer/editorial point raised, describing exactly what amendments have been made to the manuscript text and where these can be viewed (e.g. Methods section, line 12, page 5). Please also ensure that all changes to the manuscript are indicated in the text by highlighting or using track changes. If you disagree with any comments raised, please provide a detailed rebuttal to help explain and justify your decision. A point-by-point response letter must accompany your revised manuscript. This letter must provide a detailed response to each reviewer/editorial point raised, describing exactly what amendments have been made to the manuscript text and where these can be viewed (e.g. Methods section, line 12, page 5). Please also ensure that all changes to the manuscript are indicated in the text by highlighting or using track changes. If you disagree with any comments raised, please provide a detailed rebuttal to help explain and justify your decision. A point-by-point response letter must accompany your revised manuscript. This letter must provide a detailed response to each reviewer/editorial point raised, describing exactly what amendments have been made to the manuscript text and where these can be viewed (e.g. Methods section, line 12, page 5). Please also ensure that all changes to the manuscript are indicated in the text by highlighting or using track changes. If you disagree with any comments raised, please provide a detailed rebuttal to help explain and justify your decision.**

One concern regarding the classifier is related to the performance of the classifier across different cancer stages and in particular to the question whether the classification result is related to the cancer stage of the patient. In order to evaluate the performance of the classifier across cancer stages we applied a leave-one-out-cross-validation (LOOCV) classification procedure in the following way:

1. A random set of 108 cancer patients (from all cancer stages) was selected and, as explained in the paper, a dataset of 108 cancer patients and 108 patients with lung inflammation was used to developed the classifier.
2. The classifier was developed within a LOOCV procedure:
   a. Each patient is taken out from the dataset and a classifier is trained based on all remaining patients (i.e. 215 patients). Once the classifier is constructed, the patient that was taken out is classified.
   b. The procedure in (a) is repeated 216 times by which all patients are classified.
   c. Similar to the procedure reported in the paper, the PLS-LDA method is used for classification within each step in the LOOCV loop.
3. Once all patients were classified (as lung cancer or lung inflammation) the overall misclassification error, sensitivity and specificity are calculated.
4. For the lung cancer patients, the misclassification error is calculated for each cancer stage.
5. Steps 1-4 above are repeated 250 times. For each run, a random set of 108 cancer patients is selected and combined with the (fixed) set of 108 lung inflammation patients.

Note that the classification procedure described above does not take the cancer stage into account when the classifier is developed. This implies that the classifier is developed in order to discriminate between lung cancer patients and lung inflammation patients.

The table below shows the overall misclassification error, sensitivity and specificity. The results for the different cancer stages are displayed in Table 2 of the manuscript.

Overall performance (mean) of the classifier constructed on the basis of a loop of 250 leave-one-out-cross-validations (LOOCV) for different top K signature sizes.

| Top K | Misclassification | Sensitivity | Specificity |
| --- | --- | --- | --- |
| Top 12 | 12 | 90 | 86 |
| Top 16 | 11 | 90 | 87 |
| Top 20 | 11 | 90 | 88 |
